# Nonlinear Porous Diffusion Modeling of Hydrophilic Ionic Agrochemicals in Astomatous Plant Cuticle Aqueous Pores: A Mechanistic Approach

**DOI:** 10.3389/fpls.2017.00746

**Published:** 2017-05-10

**Authors:** Eloise C. Tredenick, Troy W. Farrell, W. Alison Forster, Steven T. P. Psaltis

**Affiliations:** ^1^School of Mathematical Sciences, Queensland University of TechnologyBrisbane, QLD, Australia; ^2^ARC Centre of Excellence for Mathematical and Statistical Frontiers (ACEMS), Queensland University of TechnologyBrisbane, QLD, Australia; ^3^Plant Protection Chemistry NZ Ltd.Rotorua, New Zealand

**Keywords:** uptake, plant cuticle, hydrophilic, ionic active ingredient, porous diffusion, adsorption, mathematical model, aqueous pores

## Abstract

The agricultural industry requires improved efficacy of sprays being applied to crops and weeds in order to reduce their environmental impact and deliver improved financial returns. Enhanced foliar uptake is one means of improving efficacy. The plant leaf cuticle is known to be the main barrier to diffusion of agrochemicals within the leaf. The usefulness of a mathematical model to simulate uptake of agrochemicals in plant cuticles has been noted previously in the literature, as the results of each uptake experiment are specific to each formulation of active ingredient, plant species and environmental conditions. In this work we develop a mathematical model and numerical simulation for the uptake of hydrophilic ionic agrochemicals through aqueous pores in plant cuticles. We propose a novel, nonlinear, porous diffusion model for ionic agrochemicals in isolated cuticles, which extends simple diffusion through the incorporation of parameters capable of simulating: plant species variations, evaporation of surface droplet solutions, ion binding effects on the cuticle surface and swelling of the aqueous pores with water. We validate our theoretical results against appropriate experimental data, discuss the key sensitivities in the model and relate theoretical predictions to appropriate physical mechanisms. Major influencing factors have been found to be cuticle structure, including tortuosity and density of the aqueous pores, and to a lesser extent humidity and cuticle surface ion binding effects.

## 1. Introduction

The agricultural industry, world-wide, requires improved efficacy of sprays applied to crops and weeds (Shaner and Beckie, 2014). Spray application of agrochemicals is known to be effective yet often inefficient (Knoche, 1994). There are many benefits from enhancing the efficacy of agrochemicals. Discovering attributes that increase the uptake of systemic agrochemicals can enhance efficacy and reduce the amount of active ingredient (AI) required (Balneaves et al., 1993), lower the spray water volume required, lead to more cost effective chemicals (Gaskin et al., 2013) and help minimize residues, maximize crop yield, crop quality and returns to growers (Schönherr, 2006; McKenna et al., 2013). The plant cuticle is considered the rate-limiting barrier in foliar uptake of agrochemicals (Schönherr and Riederer, 1989). Extensive research has been performed to investigate the factors involved in the mass transport of chemical compounds across the plant cuticle. However, many of the mechanisms influencing uptake are still unknown (Shaner and Beckie, 2014); the most perplexing being the differences in permeability among various plant species (Schreiber et al., 2006; Kerstiens, 2010; Forster and Kimberley, 2015). A reliable mathematical model to simulate uptake of agrochemicals would be of enormous benefit compared to performing uptake experiments, as AI uptake is specific to each AI, formulation and plant species combination, as well as environmental factors. Creating mathematical models to describe uptake should improve our understanding of the mechanisms governing the uptake of agrochemicals in plant cuticles. Zabkiewicz (2007) has noted agrochemical efficacy progress will not be made until appropriate models are created to simulate the multiple complex processes involved, creating a comprehensive agrochemical efficacy system.

Agrochemicals such as pesticides and fertilizers, along with other xenobiotics, can be either hydrophilic (ionic or uncharged) or lipophilic compounds. These two categories of AIs have opposite hydrophilicity. Lipophilic and hydrophilic compounds are governed by very different mass transport processes, which has given rise to the theory that the two types of compounds traverse the cuticle via two distinct routes (Schönherr, 2006). Hydrophilic compounds cross the cuticle via a diffusion process through aqueous pores (Baur, 1999; Schönherr, 2006). Two forms of hydrophilic compounds exist; ionic and uncharged. Ionic compounds are charged molecules that can diffuse across cuticles as they carry hydration shells (Schreiber, 2005). When ionic compounds enter the aqueous pores, they split into positive and negative ions. These ions must penetrate in equal numbers to maintain electroneutrality (Schönherr, 2006). Many plant hormones, growth regulators, plant nutrients [e.g., calcium chloride (CaCl_2_)] and pesticides, such as bentazon and glyphosate, are ionic (Schönherr and Schreiber, 2004).

Water, which is a small, uncharged but polar molecule, can use both the lipophilic and aqueous pathway within the cuticle (Schreiber et al., 2006). Lipophilic compounds penetrate the cuticle via a dissolution-diffusion process along the lipophilic pathway. Here we will focus on the uptake of hydrophilic ionic compounds (ionic AIs) across aqueous pores as this has been said to have major practical importance in the agricultural industry and significantly less is known about the permeability of the cuticle to ionic compounds than lipophilic compounds (Schreiber, 2005).

Aqueous or polar pores form preferential sites in plant cuticles for ionic compound uptake. The aqueous pathway is made up of pores of molecular dimensions filled with water (Riederer and Schreiber, 2001). Aqueous pores are nanostructures that temporarily form only when water is present (Schönherr, 2006). Cutin is a major constituent of the polymer matrix within the cuticle, which contains polar polymers. These polar polymers sorb water and swell, giving rise to aqueous pores that traverse the cuticle (Kerstiens, 2006). Estimates of average aqueous pore radii from indirect measurements are 0.3 nm in *Hedera helix* L. (Popp et al., 2005), 0.45 nm in citrus (Schönherr and Schmidt, 1979), 2.0 nm in *C. arabica* (Eichert and Goldbach, 2008), and 2.12 nm in tomato fruit cuticle membranes (CMs) (Beyer et al., 2005; Schreiber and Schönherr, 2009, p. 87).

Much work has been done to characterize the important mechanisms involved in uptake of ionic agrochemicals. Many factors, both external and internal to the cuticle, affect uptake. Yamada et al. (1964) found the adaxial and abaxial surfaces of enzymatically isolated cuticles of several plant species to be highly directionally dependent in regards to permeability to various anions and cations. When calcium ion (Ca^2+^) penetration was measured through tomato fruit cuticles, the outside to inside direction produced about 3.5 times more penetration than the inside to outside direction after 40 h. They conclude this effect is due to differences in kind, size and polarity of ions and the binding capacity of the adaxial and abaxial surfaces of cuticles. Clearly ion binding effects on cuticle surfaces have a significant impact on uptake.

Water can travel through the cuticle as free molecules in aqueous pores lined with dipoles and/or fixed ionic charges and attach to these dipoles and/or fixed charges as a monolayer (Luque et al., 1995; Kerstiens, 2006). The water content of the cuticle increases with increasing relative humidity, causing the cuticle to swell (Schönherr, 2006). The timescale for this swelling is unknown. Permeability of cuticles to ionic compounds is highly affected by relative humidity; high penetration generally occurs at high relative humidity. Relative humidity impacts the rate of surface spray droplet evaporation, which in turn affects the uptake (Ramsey et al., 2005). High relative humidity increases the number and radius of aqueous pores, which in turn facilitates the transport of ionic compounds (Middleton and Sanderson, 1965; Schönherr and Schmidt, 1979; Schönherr, 2000, 2002). Schönherr (2002) found ionic glyphosate salts with surfactants penetrated 5–10 times faster when relative humidity was increased from 70 to 100%. The same work also shows a log-linear relationship when the log of the cumulative uptake is plotted against time. This relationship represents the process whereby the aqueous pores initially take up progressively more AI until such time that the concentration of AI in the pores approaches a maximum cumulative uptake, causing the rate of uptake to slow. However, deviations from a log-linear uptake relationship were observed by Schönherr (2000) on studies using CaCl_2_. No general conclusions were made.

In summary, it has been shown experimentally that significant factors affecting the diffusion of ionic compounds include relative humidity, adjuvant, plant species, concentration of AI and ion binding capacities of cuticle surfaces (Yamada et al., 1964; Schönherr, 2000; Buchholz, 2006).

Several mathematical models on uptake through plant tissues are present in the literature. Comprehensive model reviews have been published in Forster et al. (2004) and Trapp (2004). Some models incorporate diffusion (Hsu, 1983; Satchivi et al., 2000b; Veraverbeke et al., 2003; Mercer, 2007; Pecha et al., 2012), while others employ empirical expressions (Forster et al., 2006; Schreiber and Schönherr, 2009, p. 132). Empirical models are limited to their specific measurement conditions. The formulation presented in Schreiber and Schönherr (2009) requires a single rate constant to be measured specific to each different plant species, hydrophilic AI, adjuvant and environmental conditions. Mercer (2007) has developed a diffusion model to simulate hydrophilic AI uptake through plant leaves with surfactants. They utilized a linear, cylindrical diffusion model where phloem translocation (tissue that carries nutrients to all other parts of the plant) is incorporated with a source term and no-flux boundary conditions were applied. Mercer focused on the size of the droplets on the surface and spread area effects with surfactant. No validation with experimental results was presented. Pecha et al. (2012) have developed a model for the uptake of ionic biostimulant through whole leaves by application through immersion as opposed to spraying. Their 1-D model consists of an evaporation formulation for the surface solution coupled to linear diffusion within the leaf proper and a no-flux, inner, symmetric boundary condition. The model does not incorporate porosity within the plant leaf aqueous pores. They apply their model to investigate the effect that the evaporation rate of the surface solution has on uptake. They have utilized a thin film model for evaporation, but we would be interested in droplet evaporation, which is governed by different mechanisms (Tang et al., 1997). No validation of their model predictions with experimental data is included. Satchivi et al. have produced a model in several parts (Satchivi et al., 2000a,b, 2001, 2006). They formulate a dynamic, compartment-type, nonlinear model for whole plant uptake from foliage-applied agrochemicals. The whole plant model accounts for uptake within the cuticle, leaf, stem and root in 19 compartments. Forster et al. (2004) noted that the number of experimental inputs was so vast that obtaining realistic values may not be possible.

In this work we develop a mathematical model and numerical simulation for the uptake of ionic agrochemicals through aqueous pores in plant cuticles. The model accounts for important biological and chemical mechanisms involved in uptake through aqueous pores, not previously incorporated in the modeling of agrochemical uptake. Specifically, we account for the formation and swelling of aqueous pores as a result of water uptake in the cuticle, ion binding effects and the evaporation of the spray droplet on the cuticle surface. We seek to validate the predictions of our model against available experimental results from the literature. We then discuss the key sensitivities within the model and relate this behavior to appropriate physical conditions.

## 2. Model framework

To formulate a mathematical model for uptake we need to consider the experimental setup used. The solution to the model will be verified against well-established data collected from such an experimental setup. To this end, we consider the experimental setup of Kraemer et al. (2009), who study the uptake of CaCl_2_ from droplets, containing no surfactant, applied to the surface of astomatous, isolated tomato fruit (*Solanum lycopersicum* L., cultivar “Panovy”) cuticles. Experimentally, droplets of a solution of known concentration and volume were applied to the adaxial surface of the isolated CM. In close contact with the abaxial surface of the CM was a water bath, acting as a receiver for the penetrated AI, which was analyzed for CaCl_2_ at regular time intervals after application. Kraemer et al. (2009) found that as both the initial applied concentration and time increases, then so does the penetrated amount of calcium.

**Figure 1:**
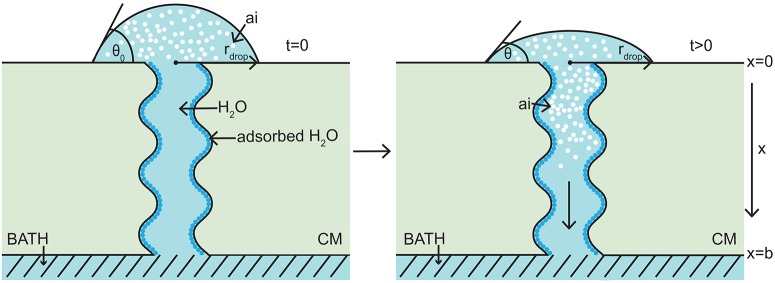
**1-D porous cuticle model domain of AI diffusion and water adsorption-diffusion**. The image on the left shows the initial conditions of the model and the image on the right a short time later. Diffusion of AI starts at the upper surface (at *x* = 0), where a drop of solution containing AI and water having initial contact angle θ_0_ and radius *r*_drop_ sits. Over time AI travels through the porous CM to the well stirred water bath at the lower surface (at *x* = *b*). Water adsorbs to the surface of the pore (shown as dark blue circles). For simplicity, a single aqueous pore can be seen crossing the cuticle (not to scale).

**Figure 2:**
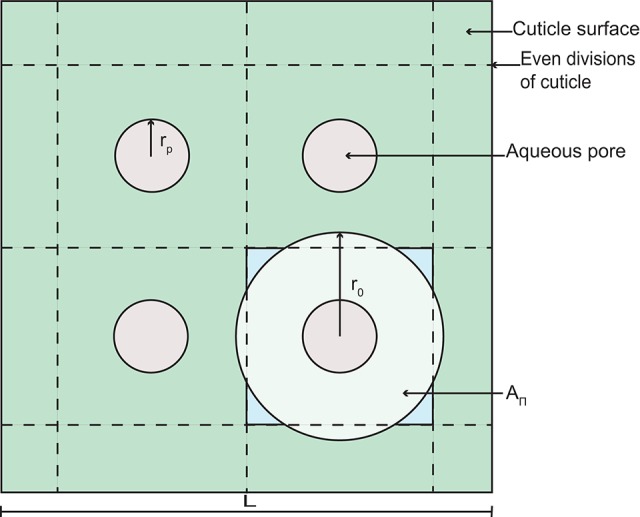
**A schematic diagram of a section of cuticle surface containing aqueous pores**. The geometry allows the calculation of the radius, *r*_0_.

The model takes the form of a nonlinear porous two-component diffusion model. A schematic diagram of the model domain considered here, in relation to the Kraemer et al. (2009) experimental setup, is shown in Figure 1, where both water and AI are diffusing. Figure 1 shows the initial conditions on the left and a short time later on the right. We consider a quasi-one-dimensional spatial domain, where all variables change primarily along the cuticular membrane thickness, *x* (0 ≤ *x* ≤ *b*). A water droplet, having initial contact angle, θ_0_, radius, *r*_drop_, and containing a known concentration of AI (CaCl_2_) sits on the adaxial surface of the cuticle, at *x* = 0. A well stirred water bath exists at the abaxial surface, at *x* = *b*. Water adsorbs to the surface of the aqueous pores and can be seen in Figure 1 as dark blue circles. For simplicity, in Figure 1, a single pore is depicted crossing the cuticle. The change of AI concentration within the droplet, at *x* = 0, and the flux of AI into the water bath, at *x* = *b*, take into account that the droplet will cover many pores. However, we are assuming that all pores can be modeled by considering what happens in a single pore and then scaling by the appropriate pore density. Further discussion of this is given around Figure 2.

Fickian diffusion is the transport mechanism ionic AI uses to traverse the plant CM aqueous pores (Schreiber and Schönherr, 2009). Diffusion of ionic AI is known to depend on the swelling of the aqueous pores (Kerstiens, 2006). Aqueous pores change in size based on adsorbed water, therefore porosity of the cuticle must be modeled. The porosity of the aqueous pores acts as a limiter to diffusion, both on the surface and through the CM.

Water facilitates the diffusion of AI. Water enters the CM and opens up the aqueous pores by attaching to the pore walls, forming an adsorption monolayer. As the pores open, water can also diffuse through the pores and act as a solvent for the mass transport for AI. Water molecules that are adsorbed onto aqueous pore walls are not available for diffusion.

The model makes the following assumptions: relative humidity and room temperature (T) are constant with values equal to those given experimentally by Kraemer et al. (2009); only water evaporates from the drop; droplet evaporation does not involve convection in liquid or gas phases; the water bath is well stirred. The AI solution is assumed to be homogeneously distributed over the surface of the CM. It is necessary to model the porosity of the cuticle as the change in porosity could be significant, therefore the aqueous pore radius needs to be modeled at every point in space and time. Pores may be very tortuous, traversing the thin cuticle laterally in lamellate like structures. The model accounts for this via the introduction of diffusivity functions that depend on porosity and tortuosity.

The variables and parameters with their associated units and references (where possible) are described in Table 1. The model, including the governing partial differential equations (PDEs), initial conditions (ICs), boundary conditions (BCs), and auxiliary functions is as follows:

PDEs:

(1) $$\begin{array}{l} \frac{\partial (\varepsilon c_{\text{AI}})}{\partial t}=-\frac{\partial }{\partial x}\left[-D_{\text{AI}}\left(\frac{\partial (\varepsilon c_{\text{AI}})}{\partial x}\right)\right],\\ \;0<x<b,\;t>0, \end{array}$$

(2) $$\begin{array}{l} \frac{\partial (\varepsilon c_{{\text{H}}_{2}\text{O}})}{\partial t}=-\frac{\partial }{\partial x}\left[-D_{{\text{H}}_{2}\text{O}}\left(\frac{\partial (\varepsilon c_{{\text{H}}_{2}\text{O}})}{\partial x}\right)\right]-\frac{2}{r_{\text{p}}}(1-\varepsilon )\frac{\partial \Gamma_{{\text{H}}_{2}\text{O}}}{\partial t},\\ \;0<x<b,\;t>0, \end{array}$$

Functions:

(3) $$\begin{array}{l} \Gamma_{{\text{H}}_{2}\text{O}}(x,t)=\frac{\Gamma_{\text{S}}\;\beta_{{\text{H}}_{2}\text{O}}\;c_{{\text{H}}_{2}\text{O}}}{1+\beta_{{\text{H}}_{2}\text{O}}\;c_{{\text{H}}_{2}\text{O}}},\\ \;0<x<b,\;t>0, \end{array}$$

(4) $$\begin{array}{l} r_{\text{p}}(x,t)\;=r_{{\text{H}}_{2}\text{O}}\left[1+{\left(\sin\left({\left(\Gamma_{{\text{H}}_{2}\text{O}}\;r_{{\text{H}}_{2}\text{O}}^{2}\;N_{\text{A}}\right)}^{-1}\right)\right)}^{-1}\right],\\ \;0<x<b,\;t>0, \end{array}$$

(5) $$\begin{array}{l} \varepsilon (x,t)\;=\;\pi {\left[\frac{r_{\text{p}}}{L}\left(\sqrt{n_{0}}+1\right)\right]}^{2},\\ \;0<x<b,\;t>0, \end{array}$$

(6) $$\begin{array}{l} D_{\text{AI}}(x,t)=\;D_{\text{AI}}^{\text{bulk}}\;\varepsilon^{\left(\frac{F_{\text{s}}}{2-F_{\text{s}}}\right)},\\ \;0<x<b,\;t>0, \end{array}$$

(7) $$\begin{array}{l} D_{{\text{H}}_{2}\text{O}}(x,t)=\;D_{{\text{H}}_{2}\text{O}}^{\text{bulk}}\;\varepsilon^{\left(\frac{F_{\text{s}}}{2-F_{\text{s}}}\right)},\\ \;0<x<b,\;t>0, \end{array}$$

ICs:

(8) $$c_{\text{AI}}(x,0)=0,\;0<x<b,$$

(9) $$c_{\text{AI}}(0,0)=c_{\text{AI},0}^{\text{drop}},$$

(10) $$r_{\text{p}}(x,0)=r_{\text{p}}^{\text{max}}\;H,\;0\leq x\leq b,$$

(11) $$c_{{\text{H}}_{2}\text{O}}(x,0)=c_{{\text{H}}_{2}\text{O}}^{\text{pure}},\;0<x<b,$$

(12) $$c_{{\text{H}}_{2}\text{O}}(0,0)=\frac{1-{\bar{v}}_{\text{AI}}c_{\text{AI}}(0,0)}{{\bar{v}}_{{\text{H}}_{2}\text{O}}},$$

(13) $$\begin{array}{l} \Gamma_{{\text{H}}_{2}\text{O}}(x,0)={\left(r_{{\text{H}}_{2}\text{O}}^{2}\;N_{A}\arcsin\left({\left(\frac{r_{\text{p}}(x,0)}{r_{{\text{H}}_{2}\text{O}}}-1\right)}^{-1}\right)\right)}^{-1},\\ \;0<x<b, \end{array}$$

(14) $$\begin{array}{l} \beta_{{\text{H}}_{2}\text{O}}={\left(c_{{\text{H}}_{2}\text{O}}(x,0)\left[\frac{\Gamma_{\text{S}}}{\Gamma_{{\text{H}}_{2}\text{O}}(x,0)}\;-1\right]\right)}^{-1},\\ \;0<x<b, \end{array}$$

BCs - AI (drop):

(15) $$\begin{array}{l} \frac{\partial }{\partial t}\left[V_{{\text{H}}_{2}\text{O}}^{\text{drop}}(t)c_{\text{AI}}(0,t)\right]=-kc_{\text{AI}}(0,t)+\rho_{0}A_{\text{drop}}A_{\Pi }D_{\text{AI}}(0,t)\\ \;{\frac{\partial }{\partial x}\left[\varepsilon (x,t)c_{\text{AI}}(x,t)\right]|}_{x\;=\;0}, \end{array}$$

Functions:

(16) $$\begin{array}{l} V_{{\text{H}}_{2}\text{O}}^{\text{drop}}(t)\\ =\{\begin{array}{ll} \frac{\delta r_{\text{drop}}^{3}}{{\left[\frac{V_{0}}{\delta r_{\text{drop}}^{3}+\frac{V_{0}}{4}}-\frac{2D_{\text{evap}}\psi t}{r_{\text{drop}}^{2}\rho_{\text{L}}}\right]}^{-1}-\frac{1}{4}} & \;:\;V_{{\text{H}}_{2}\text{O}}^{\text{drop}}(t)>V_{\infty },\\ V_{\infty }\; & :\;V_{{\text{H}}_{2}\text{O}}^{\text{drop}}(t)\leq V_{\infty }, \end{array} \end{array}$$

(17) $$\psi =\frac{M_{\text{w}}P_{\text{v}}}{RT}(1-H),$$

(18) $$r_{\text{drop}}=\;{\left(\frac{3V_{0}}{\pi g(\theta_{0})}\right)}^{\frac{1}{3}}\sin(\theta_{0}),$$

(19) $$A_{\text{drop}}=\;\pi r_{\text{drop}}^{2},$$

(20) $$g(\theta_{0})={\left(1-\cos\theta_{0}\right)}^{2}(2+\cos\theta_{0}),$$

(21) $$\text{BC - AI (bath)}:c_{\text{AI}}(b,t)=0,\;t>0,$$

(22) $${\text{BC-H}}_{2}\text{O}(\text{drop}):c_{{\text{H}}_{2}\text{O}}(0,\text{t})\text{=}\frac{1-{\bar{v}}_{\text{AI}}c_{\text{AI}}(0,\text{t})}{{\bar{v}}_{{\text{H}}_{2}\text{O}}},\text{ t}>0,$$

(23) $${\text{BC-H}}_{2}\text{O}(\text{bath}):c_{{\text{H}}_{2}\text{O}}(\text{b},\text{t})=c_{{\text{H}}_{2}\text{O}}^{\text{pure}},\text{ t}>0.$$

**Table 1:** **Model parameters**.

**Parameter**	**Definition**	**Value and units**	**Comments**
*A*_Π_	Circular cross sectional area of control volume cylinder	m^2^	refer to Section 2.2
*A*_drop_	Drop surface contact area	m^2^	Surface contact area of drop on cuticle surface, Erbil et al. (2002)
AI	Active ingredient		
*b*	Thickness of cuticle	1.87*e*−5 m	Chamel et al., 1991
BC	Boundary condition		
$c_{\text{AI},0}^{\text{drop}}$	Concentration of AI in drop at *t* = 0	mol/m^3^	Kraemer et al., 2009
$c_{{\text{H}}_{2}O}^{\text{pure}}$	Pure water concentration at 20^0^ C and *t* = 0	55,409.78 mol/m^3^	calculated
*c*_*i*_(*x, t*)	Concentration of component *i* in plant cuticle	mol/m^3^	
CM	Cuticle membrane		
$D_{\text{AI}}^{\text{bulk}}$	Self/bulk diffusion coefficient of AI	7.93*e*−10 m^2^/s	For CaCl_2_, Ca^2+^ diffuses the slowest, so Ca^2+^ value is used, Yuan-Hui and Gregory (1974)
$D_{{\text{H}}_{2}O}^{\text{bulk}}$	Self/bulk diffusion coefficient of water	2.299*e*−9 m^2^/s	Holz et al., 2000
*D*_evap_	Diffusivity of water in air	2.4*e*−5 m^2^/s	Semenov et al., 2013
*D*_*i*_	Diffusivity of component *i*	m^2^/s	Liu and Nie, 2001
*F*_s_	Fractal scaling dimension	1.1 (-)	1 < *F*_*s*_ < 2 (fitted, refer to Section 2.7)
*H*	Relative humidity	0.7 (70%)	Kraemer et al., 2009
*i*	Component AI (CaCl_2_) or H_2*O*_		
IC	Initial condition		
*k*	Ion binding reaction rate constant	1.07*e*−15 m^3^/s	(fitted, refer to Section 2.7)
*L*	Control volume length	1 m	
*M*_w_	Molecular weight H_2_O	0.018015 kg/mol	
*N*_A_	Avogadro constant	6.02214*e*23 mol^−1^	
*n*_0_	Number of aqueous pores on 1 m^2^ of cuticle	(-)	Refer to Section 2.2
*P*_v_	Saturated water vapor pressure in air at 20^0^ C	2338.8 Pa	Lide, 2004
PDE	Partial differential equation		
R	Gas constant	8.3145 Pa·m^3^/K/mol	
*r*_drop_	Droplet contact radius	m	Contact radius of drop on cuticle surface, calculated in Equation (18), Erbil et al. (2002)
*r*_H_2*O*__	Van der Waals radius of a water molecule	1.5*e*−10 m	Schreiber et al., 2006
*r*_p_(*x, t*)	Radius of aqueous pore	m	
$r_{\text{p}}^{\text{max}}$	Maximum radius of aqueous pores	2.12*e*−9 m	For tomato fruit cuticle, (Schreiber and Schönherr, 2009, p. 87)
*t*	Time	s	
*T*	Temperature	293.15 K	Kraemer et al., 2009
*V*_0_	Volume of droplet at *t* = 0	1*e*−9 m^3^	Kraemer et al., 2009
${\bar{v}}_{\text{AI}}$	Partial molar volume CaCl_2_	1.6*e*−5 m^3^/mol	Oakes et al., 1995
${\bar{v}}_{{\text{H}}_{2O}}$	Partial molar volume water	1.8047*e*−5 m^3^/mol	Zen, 1957
*V*_∞_	Smallest volume of droplet	m^3^	Refer to Section 2.4
*x*	Length	m	
β_H_2*O*__	Langmuir parameter	3.77*e*−5 m^3^/mol	Equilibrium parameter of adsorbed water (calculated in Equation 14).
δ	Evaporation constant	1.994	Schönfeld et al., 2008
ε(*x, t*)	Porosity of cuticle	(-)	0 < ε < 1
Γ_H_2*O*__(*x, t*)	Concentration of water adsorbed per unit area at equilibrium	mol/m^2^	Luque et al., 1995; Bard and Faulkner, 2001
Γ_S_	Langmuir saturation constant	9.6832*e*−4 mol/m^2^	0 < Γ_H_2*O*__ < Γ_S_, saturation concentration of water adsorbed in aqueous pores per unit area (calculated in Section 2.2)
ψ	Saturated water vapor concentration with relative humidity	kg/m^3^	Erbil, 2012
ρ_0_	Density of aqueous pores in cuticle	8.4*e*14 m^−2^	(fitted, refer to Section 2.7)
ρ_L_	Liquid density H_2_O at 20^0^ C	998.2071 kg/m^3^	Weast and Lide, 1989
θ_0_	Contact angle of drop on cuticle surface at *t* = 0	1.7314 rad (99.2^*o*^)	For CaCl_2_ (no data for tomato fruit cuticle), Schmitz-Eiberger et al. (2002)

*Dimensionless parameters are shown in Section 2.6 and Equation (A-1)*.

### 2.1. Governing equations

Here the governing equations will be described, then in Section 2.2, the auxiliary functions will be explained in further detail. The concentration of AI, *c*_AI_, is governed by diffusion, as shown in Equation (1). This equation is nonlinear. It couples to Equation (2) via the fact that both the diffusivity of AI, *D*_AI_ and the porosity of the cuticle, ε, are functions of the water concentration in the cuticle, *c*_H_2*O*__. These dependencies are given explicitly in Equations (3–7).

The concentration of water within the pore, *c*_H_2_O_, is governed by diffusion and reaction, shown in Equation (2). Both transport and reaction are nonlinear. The reaction is governed by the time rate of change of the concentration of water that adsorbs to the surface of the pores, Γ_H_2_O_.

### 2.2. Auxiliary functions

The concentration of water molecules that adsorb to the pore walls per unit area, Γ_H_2_O_, is determined from a Langmuir isotherm (Giles et al., 1974; Luque et al., 1995; Bard and Faulkner, 2001) and is given in Equation (3). It is proportional to the concentration of water adjacent to the pore wall, *c*_H_2_O_, the likelihood that a water molecule will bind to the pore wall, β_H_2_O_, and the maximum possible (or saturated) concentration of adsorbed water that can be supported by the pore surface, Γ_S_.

Equation (4) describes how aqueous pores change in size due to water adsorption. A given aqueous pore radius, *r*_p_, changes based on the radius of a water molecule, *r*_H_2_O_, and the concentration of adsorbed waters per unit area, Γ_H_2_O_. Equation (4) can be found with a simplistic formulation as follows. If we consider a single cylindrical pore and assume that at every point in space (through the cuticle) it has a circular cross-section, then the size of this circle depends on the number of water molecules, here assumed to be spheres, adsorbed in a monolayer on the pore surface. The number of water molecules adsorbed, *n*_H_2_O_, is found by multiplying Γ_H_2_O_ by the area occupied by a water molecule on the pore wall, thus:

(24) $$n_{{\text{H}}_{2}\text{O}}=\Gamma_{{\text{H}}_{2}\text{O}}\;\pi \;r_{{\text{H}}_{2}\text{O}}^{2}\;N_{\text{A}}.$$

Assuming a geometric arrangement of water molecules that is given by a closed Steiner chain (Maor and Jost, 2014; Caglayan, 2016, pp. 130–133), we may write that:

(25) $$\sin\left(\frac{\pi }{n_{{\text{H}}_{2}\text{O}}}\right)=\frac{r_{{\text{H}}_{2}\text{O}}}{r_{\text{p}}-r_{{\text{H}}_{2}\text{O}}}.$$

Substituting Equation (24) into Equation (25) and solving for *r*_p_, we then obtain Equation (4).

The formulation in Equation (4) can be used to find the constant describing the saturated concentration of water molecules adsorbed to the aqueous pore walls per unit area, Γ_S_. This is done by assuming the pore radius is at its maximum when the pore surface is fully saturated and substituting $r_{\text{p}}=r_{\text{p}}^{\text{max}}$ and Γ_H_2_O_ = Γ_S_ into Equation (4) and then solving for Γ_S_.

Porosity, ε, limits diffusion of AI and water through the cuticle. In Equation (5), porosity changes in space and time based on the aqueous pore radius, *r*_p_, the length, *L* (defined below), and the number of aqueous pores in the cuticle, *n*_0_. To formulate ε, we assume that pores are evenly distributed on the surface of the cuticle. Furthermore, at any point through the cuticle, we assume that the pore has a circular cross section of area $\pi r_{\text{p}}^{2}$. Given this, each pore has associated with it a volume of cuticle with radius, *r*_0_, which at any point through the cuticle, has a circular cross section of area $\pi r_{0}^{2}$. The porosity at any point through the cuticle is then given by:

(26) $$\varepsilon (x,t)=\frac{\pi r_{\text{p}}^{2}}{\pi r_{0}^{2}}={\left(\frac{r_{\text{p}}}{r_{0}}\right)}^{2}.$$

To determine *r*_0_ in Equation (26), we assume that a square area (*L*^2^ m^2^) of cuticle can be divided evenly into square subdivisions, as shown in Figure 2. At the center of each subdivision, we have an aqueous pore opening (this is consistent with our earlier assumption that pores are evenly distributed on the surface of the cuticle). The number of pores on the area *L*^2^ is given by $n_{0}=\rho_{0}\;L^{2}$, where ρ_0_ is the initial aqueous pore density. In Figure 2, *n*_0_ = 4, for example. We now assume that the circular cross section of cuticle, which is associated with each aqueous pore (see our earlier assumption) has an area, *A*_Π_, that is equal to the area of our square subdivision, as shown in Figure 2. Given this, we may write that:

$$\pi r_{0}^{2}={\left(\frac{L}{\sqrt{n_{0}}+1}\right)}^{2},$$

which can be rearranged for *r*_0_ and substituted into Equation (26), yielding Equation (5).

The diffusivities of AI and water are given in Equations (6) and (7), respectively. They are functions of the bulk diffusion coefficients, $D_{\text{AI}}^{\text{bulk}}$ and $D_{{\text{H}}_{2}\text{O}}^{\text{bulk}}$, porosity and tortuosity as described by the fractal scaling dimension of the aqueous pores, *F*_s_. A function of ε(*x, t*) is used here instead of a diffusion constant, as diffusivity changes as the size and shape of the pores vary. The tortuosity is incorporated here as a fractal scaling dimension, *F*_s_. Here *F*_s_ is limited to 1 < *F*_s_ < 2, where higher values describe more tortuous pores. This approach has been noted in the literature as being superior to other tortuosity formulations (Liu and Nie, 2001; Yuan and Sundén, 2014).

### 2.3. Initial conditions

The initial conditions of the model are given in Equations (8–14). Equation (8) states that there is no AI in the CM aqueous pores initially. Equation (9) states that there is initially a constant concentration of AI applied in the droplet solution, $c_{\text{AI},0}^{\text{drop}}$. The initial pore radius can be calculated by $r_{\text{p}}^{\text{max}}\times H$ in Equation (10). The relative humidity, *H*, appears here as it is known that the pore radius is significantly affected by humidity (Schönherr and Schmidt, 1979; Schönherr, 2000).

Equation (11) states that there is initially a constant concentration of water in the pores, $c_{{\text{H}}_{2}\text{O}}^{\text{pure}}$. In the Kraemer et al. (2009) experimental setup, cuticles are rehydrated initially, so in the model it is assumed that the pores are initially filled with water.

Equation (12) is simply Equation (22) at *t* = 0. Equation (22) is described in Section 2.4. The initial values for Γ_H_2_O_ and β_H_2_O_ in Equations (13) and (14) are found by rearranging Equations (3) and (4).

### 2.4. Boundary conditions

The cuticle surface mechanisms involved in uptake are significant. Therefore, it is important to incorporate within the model effects such as evaporation due to environmental conditions and the ion binding capacity of the cuticle surface (Yamada et al., 1964; Semenov et al., 2013). The BCs in Equations (15–20) govern the change in AI concentration on the adaxial surface of the CM within the drop. Only water evaporates from the drop. Hence only the change in the volume of water, $V_{{\text{H}}_{2}\text{O}}^{\text{drop}}\left(t\right)$, is modeled. In Equation (15), as water evaporates from the drop the concentration of AI in the drop, *c*_AI_, increases (left hand side of equation), then as the AI is transported from the drop into the CM (right hand side of equation) the concentration of AI in the drop decreases, governed by the circular cross sectional area of the control volume cylinder, *A*_Π_, diffusivity of AI, *D*_AI_, number of pores under the drop, ρ_0_ *A*_drop_, and porosity of the cuticle, ε.

Ions are bound to the cuticle surface and lost to diffusion (Yamada et al., 1964). This is incorporated into Equation (15) using a reaction rate constant, *k*. If *k* is a non-zero number, the total percentage uptake of AI cannot reach 100%. A constant value for *k* is appropriate here, as temperature is not varied experimentally.

The evaporation of water is formulated in Equation (16) based on a constant radius of the droplet, *r*_drop_, initial volume of the droplet, *V*_0_, diffusivity of water in air, *D*_evap_, liquid density of water, ρ_L_, molecular weight of water, *M*_W_, saturated water vapor pressure, *P*_v_, temperature, *T*, and relative humidity, *H*. Evaporation can be simulated using a sessile drop model and spherical-cap geometry (Erbil, 2012). Semenov et al. (2013) have noted that much is unknown about evaporation of complex fluids such as surfactant solutions. However, they conclude evaporation in still air generally occurred in four stages. For the majority of the time only two stages governed evaporation; constant contact angle or constant contact radius mode. We will exclusively use constant contact radius mode for simplicity. Using constant contact radius mode alone will allow us to later scale with a constant droplet area to calculate uptake, described in Section 2.5. The evaporation model used here in Equations (16–20) is derived in Schönfeld et al. (2008) and Erbil (2012).

Schönherr (2000) observed that when CaCl_2_ is applied to isolated cuticles, the salt residue on the surface during uptake appeared as transparent liquid phases and crystals were not seen, due to the very low point of deliquescence of CaCl_2_. In fact it will remain in solution unless relative humidity is below 32% at 20^*o*^C (Kolthoff et al., 1969; Tang et al., 1997; Dow, 2003)^1^. Equation (16) will produce a negative volume of water, $V_{{\text{H}}_{2}\text{O}}^{\text{drop}}$, after long times. To overcome this, in Equation (16), the volume of water, $D_{{\text{H}}_{2}\text{O}}^{\text{drop}}$, is set to a constant, *V*_∞_, which represents the minimum volume of water left on the cuticle surface at longer times. The constant *V*_∞_ can be calculated based on the known solubility of CaCl_2_ in terms of concentration at 20 ^*o*^C (Stephen et al., 1963), and noting that $c_{\text{AI},0}^{\text{drop}}$ $V_{0}=c_{\text{AI}}^{\text{sat}}V_{\infty }$. In terms of the Kraemer et al. (2009) data, we calculate that *V*_∞_ = 1.34*e*−12, 6.71*e*−12, 1.34*e*−11, 2.01*e*−11, 4.03*e*−11 m^3^ for each corresponding $c_{\text{AI},0}^{\text{drop}}$ of 9.01, 45.05, 90.1, 135.2, 270.3 mol/m^3^. Under this formulation, the concentration of AI in the drop cannot exceed the solubility concentration, $c_{\text{AI}}^{\text{sat}}$, as the volume of the drop has reached a constant, *V*_∞_.

To account for different types of adjuvants, the initial droplet contact angle, θ_0_, can be varied. Schmitz-Eiberger et al. (2002) found that for CaCl_2_ on parafilm without surfactant the contact angle was 99.2^0^ and for CaCl_2_ with the surfactant RSO 5, the contact angle decreased to 76.8^0^.

The BC for AI at the bath is shown in Equation (21). The concentration of AI, *c*_AI_, is zero as the solution is well stirred at the abaxial surface of the cuticle.

Equation (22) is a conservation of volume statement, where the volume of the drop is given by the sum of the volume of AI in the drop and volume of water in the drop. Then we can substitute $\varepsilon_{\text{AI}}^{\text{drop}}=V_{\text{AI}}^{\text{drop}}/V_{\text{total}}^{\text{drop}}$ and $\varepsilon_{{\text{H}}_{2}\text{O}}^{\text{drop}}=V_{{\text{H}}_{2}\text{O}}^{\text{drop}}/V_{\text{total}}^{\text{drop}}$ into the conservation of volume statement, producing $\varepsilon_{\text{AI}}^{\text{drop}}+\varepsilon_{{\text{H}}_{2}\text{O}}^{\text{drop}}=1$. Then we can substitute into the previous equation $\varepsilon_{\text{AI}}^{\text{drop}}={\bar{v}}_{\text{AI}}c_{\text{AI}}$ and $\varepsilon_{\text{AI}}^{\text{drop}}={\bar{v}}_{\text{AI}}c_{\text{AI}}$ producing Equation (22).

### 2.5. Uptake calculation

The output of our model is the concentration of AI and water through the entire CM. However, experiments “measure uptake or penetration,” which is the cumulative mass of AI in the water bath. Therefore, we need to convert our model's output to compare to experimental results. The calculation of the final uptake or penetration of Ca, *M*_*t*_ (in μg), at the water bath for comparison with Kraemer et al. (2009) is as follows:

(27) $$\begin{array}{l} M_{t}=\;10^{6}\;M_{\text{w},{\text{CaCl}}_{2}}\;\rho_{0}\;A_{\text{drop}}\;A_{\Pi }\;n_{\text{drops}}\;\overset{t_{\text{final}}}{\underset{0}{\int }}\\ \;{-D_{AI}\left(\frac{\partial (\varepsilon c_{AI})}{\partial x}\right)|}_{x\;=\;b}\;.dt. \end{array}$$

Here the constant 10^6^ converts from g to μg, *M*_w, CaCl_2__ is the molecular weight of CaCl_2_ (110.98 g/mol) and *n*_drops_ is the number of individual drops applied to the cuticle surface (5 in the Kraemer et al. (2009) experiment). The flux at the bath boundary is integrated over time, where *t*_final_ is the experiment duration (48 × 60 × 60 s in Kraemer et al. (2009)).

### 2.6. Dimensionless model

The nonlinear, plant cuticle diffusion model as described in Equations (1–23) can be scaled and simplified using dimensionless parameters. This allows a sensitivity analysis to be performed, which is discussed in Section 3.1. For completeness, the full dimensionless model is shown in Appendix A of Supplementary Material. The following dimensionless parameters were used:

(28) $$\bar{F}=\frac{F_{s}}{2-F_{s}},$$

(29) $$\bar{\gamma }=\frac{\rho_{0}\;A_{\text{drop}}\;A_{\Pi }\;b}{V_{0}}.$$

The dimensionless parameter $\bar{F}$, shown in Equation (28), is based on the fractal scaling dimension, *F*_*s*_, used in the diffusivity function in Equation (A-7). The parameter $\bar{\gamma }$ describes the volume of pores through the cuticle, shown in Equation (29). It is the ratio of the number of pores, ρ_0_ *A*_drop_, area of the pore control area, *A*_Π_, and thickness of the cuticle, *b*, to the initial droplet volume, *V*_0_. The parameter $\bar{\gamma }$ influences how much AI can diffuse into the cuticle from the surface solution in Equation (A-15).

### 2.7. Numerical solution procedure

The dimensionless, nonlinear plant cuticle diffusion model as described in Equations (A-2–A-19) is solved numerically. This is done by discretizing the model's PDEs using second order central differences to approximate the spatial derivatives and averaging of the diffusivity function at the control volume faces (Grasselli and Pelinovsky, 2008, Chapter 6). The resulting system of ordinary differential equations is then solved using “ode15i” (Shampine, 2002) within MATLAB® 2016a (MATLAB, 2016).

The values for *F*_*S*_, *k*, and ρ_0_ are described in Table 1. Kraemer et al. (2009) has studied the uptake of five different initial applied masses of Ca of 5, 25, 50, 75, and 150 μg. The parameters *F*_*S*_, *k* and ρ_0_ were found by focusing on uptake of only the initial applied mass of 150 μg of Ca. The parameters were then fitted to that data using trial-and-error. The parameters were kept constant and used to solve the uptake of the other four initial applied masses.

### 2.8. Summary of plant species accommodations within the model

Plant species variation is known to have a major effect on uptake. The variations in uptake based on differing plant species are numerous and not fully understood (Forster and Kimberley, 2015). However, several well known effects have been incorporated into the model in various ways, making novel additions to a simple diffusion model. The model incorporates plant species variation in uptake by including:

- θ_0_—the initial contact angle on the plant surface, which varies widely based on plant species (Nairn et al., 2013),
- $r_{\text{p}}^{\text{max}}$—the maximum size of aqueous pores, which is plant species specific,
- ρ_0_ & *n*_0_—density of aqueous pores varies depending on plant species,
- Γ_S_ & β_H_2_O_— Langmuir parameters, which vary depending on plant species as water adsorption varies,
- *F*_s_—tortuosity or complexity of aqueous pores varies between plant species,
- *b*—thickness of cuticle, which can vary from 0.1 to 10 μm (Holloway, 1982; Jeffree, 1996), has been shown to affect uptake where increasing thickness leads to decreasing penetration (Santier and Chamel, 1992),
- *k*—ion binding capacity of the cuticle surface, which is known to vary significantly between plant species (Yamada et al., 1964).

## 3. Results and discussion

**Figure 3:**
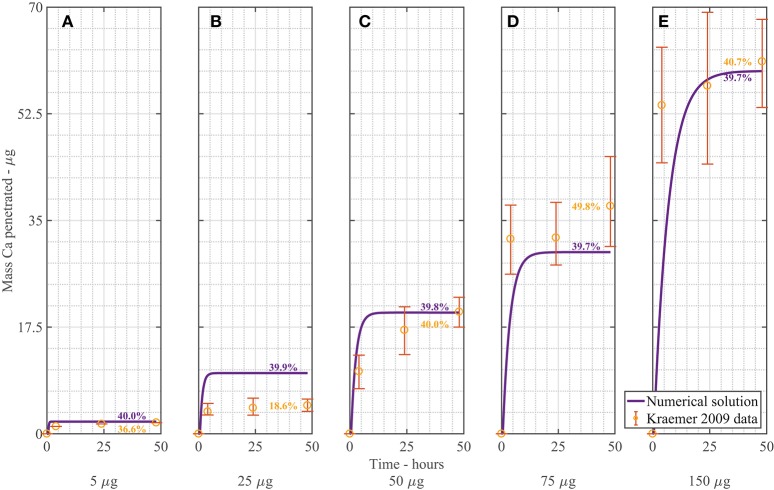
**Numerical solution of the plant cuticle diffusion model compared to experimental data from Kraemer et al. (2009) using mass of Ca applied of 5 μg (A)**, 25 μg **(B)**, 50 μg **(C)**, 75 μg **(D)**, 150 μg **(E)** of AI over 48 h with parameters outlined in Table 1. The numerical solution can be seen as the continuous purple line and the experimental data as orange circles with error bars. The final percent Ca uptake is shown on each subfigure at 48 h.

The dimensionless, nonlinear plant cuticle diffusion model as described in Equations (A-2–A-19) is solved numerically. Figure 3 shows a comparison of the numerical solution of the model compared to the experimental data from Kraemer et al. (2009). All parameters used here are shown in Table 1 and are the same for all five plots, with the exception that five different applied initial concentrations of AI are used, namely $c_{\text{AI},0}^{\text{drop}}$ = 1 (A), 5 (B), 10 (C), 15 (D), 30 g/L (E) (corresponding to a mass of 5, 25, 50, 75, 150 μg). The formulation of the mass Ca penetrated is described previously in Equation (27). The mass Ca penetrated is converted to percent Ca uptake at 48 h as shown in Figure 3, as described in Section 3.1.

Overall the numerical solutions and the experimental data in Figure 3 compare well, considering the complex mechanisms involved. We do note, however, that the most significant discrepancy between our model and the Kraemer et al. (2009) data occurs in Figure 3B. When we calculate the total uptake in each of the (Figures 3A,C–E) as a percentage of the initial applied mass of Ca in the droplets, we find that the mean percentage uptake is 42% with a coefficient of variation of 14%. However, in the experimental data of Figure 3B, this uptake is only 18.6%. This may be an outlier. No explanation is provided by Kraemer et al. (2009) in regards to this.

We can consider the error associated with Figure 3 when the numerical solution is compared to experimental data. We include the error bars and exclude the data in Figure 3B and find an an R^2^ value of 83.2%. This value is reasonable, given the complex mechanisms involved in the model. We do note that the original error bars in Kraemer et al. (2009) for Figure 3A were not discernible, so have been excluded.

In Figure 3, as $c_{\text{AI},0}^{\text{drop}}$ increases, the penetrated amount of Ca increases. From the figure, we can see that over the first 2–10 h, uptake occurs rapidly, then levels out and approaches a maximum value. This rapid increase in uptake initially matches the trend in the experimental data. The difference between the concentration of AI in the drop and the cuticle rapidly increases due to droplet evaporation, which in turn causes rapid initial uptake. Uptake levels out after approximately 10 h. Uptake levels out due to the concentration of AI in the drop reaching zero. The maximum AI uptake value is governed by ion binding. Ions are bound to the cuticle surface and therefore lost to diffusion. A high percentage of ions are lost to ion binding. The mean percentage uptake of the Kraemer et al. (2009) data at 48 h is 37%. This may then indicate that 63% of ions are lost to ion binding. The numerical uptake at 48 h agrees well with the Kraemer et al. (2009) data at 48 h.

**Figure 4:**
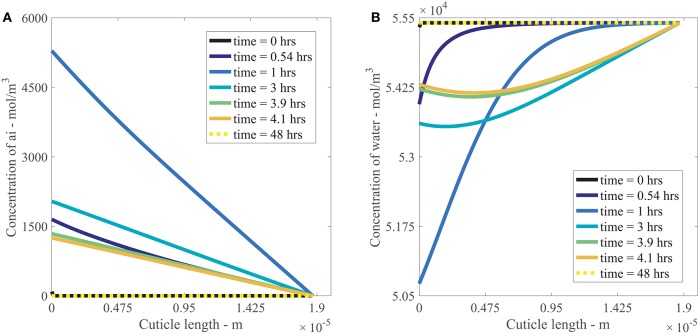
**Nonlinear plant cuticle diffusion model results for a single applied concentration of AI (A)** and water **(B)** over 48 h using parameters outlined in Table 1. The initial condition is shown as a black line and the boundary conditions for the drop and bath are located at cuticle length *x* = 0 and *x* = 1.87*e*−5 m respectively. The black solid line is shown at *t* = 0 h, and the dashed yellow line on top of that black line is at *t* = 48 h, as shown in the legend.

Figures 4A,B show the results of AI diffusion, and water diffusion with adsorption in the cuticle aqueous pores over 48 h. The output in the figures is from a single applied AI concentration of 10 g/L. In both figures, the drop is located at *x* = 0 m and the water bath is located at *x* = 1.87*e*−5 m.

In Figure 4A, we see that the concentration of AI at *x* = 0 m increases to approximately 5,000 mol/m^3^ at 1 h as the surface solution becomes more concentrated due to evaporation. Once the concentration of AI reaches its solubility concentration (discussed in Section 2.4), it ceases to increase and then decreases as the concentration of AI is transported via diffusion into the cuticle. At the final time of 48 h, the concentration of AI at *x* = 0 m has reached zero as all the AI available for diffusion has been either transported into the cuticle via diffusion or lost to ion binding on the cuticle exterior. Within the cuticle, AI is transported from regions of high concentration to low concentration via diffusion, with the most rapid change in AI concentration occurring before 4 h. This matches the timescale evident in the data from Kraemer et al. (2009). At late times, the concentration of AI has reached equilibrium and is zero everywhere in the cuticle pore.

Figure 4B shows the diffusion and adsorption of water in the aqueous pores over time. Initially the concentration of water is a constant everywhere, except at *x* = 0 m, where it is slightly lower due to the presence of AI. At *x* = 0 m, the concentration of water decreases due to evaporation. The water concentration in the cuticle decreases up to 1 h, then increases after 1 h as water diffuses back toward the drop from the water bath. At late times, the concentration of water returns to the concentration of pure water throughout the whole cuticle.

Swelling of the aqueous pores (not shown) occurs whilst uptake takes place. In the context of the Kraemer et al. (2009) experimental setup, where cuticles are rehydrated initially and a water bath is located on the lower cuticle surface, the pore swelling is not significant. We leave pore swelling in the model with the view to investigating this in future works.

The values for *F*_*S*_, *k*, and ρ_0_ are described in Table 1. Here we will investigate these parameters. A low value of *F*_S_ is obtained from the fitting exercise. A low *F*_S_ would describe a pore at the low tortuosity end of the range. If a higher value was used, the uptake would be more gradual, as the pores would then be more tortuous. If a certain plant species cuticle is known to have slow uptake, a low diffusivity, very tortuous pores or a high lamellate structure, a higher *F*_S_ can be chosen appropriately. Fitting *F*_*s*_ is reasonable as this facilitates the calculation of the diffusion path length. The diffusion path length through plant cuticles cannot currently be established by a physical measurement (Riederer and Schreiber, 1995). Yuan and Sundén (2014) have provided values of 1.1 ≤ *F*_s_ ≤ 1.3 in porous structures to use as reference points. Therefore, our fitted value of *F*_*s*_ = 1.1 is reasonable. The value for ρ_0_ in Table 1 closely agrees with values for ρ_0_ found elsewhere ranging from 5.1*e*13 m^−2^ in *Populus x canescens* (Aiton) Sm. leaves (Remus-Emsermann et al., 2011) to 2*e*15 m^−2^ in *Citrus aurantium* cuticles (Schreiber and Schönherr, 2009, p. 85). Therefore, our fitted value for ρ_0_ would seem to be reasonable.

This model can be theoretically applied to the uptake of most ionic hydrophilic AI without adjuvants. It cannot apply to uncharged hydrophilic compounds, ionic compounds which would dehydrate pores such as Fe chelates (Schönherr et al., 2005; Schlegel et al., 2006) or lipophilic compounds. The model can also theoretically be applied to any isolated astomatous plant leaf or fruit species cuticle, where the aqueous pores are sufficiently large to allow AI to be transported by Fickian diffusion. If ionic AI penetrates through a certain plant species at a very slow rate, transport is theorized to be a mechanism alternate to Fickian diffusion and this situation would require further investigation before the model could be applied.

Overall the validation results of our plant cuticle model with the Kraemer et al. (2009) data in Figure 3 shows good agreement with the experimental data, confirming that our model can be appropriately applied to uptake experiments, simulating the important governing mechanisms. This model provides a good basis for future work.

### 3.1. Sensitivity analysis

A sensitivity analysis was performed with the results from the dimensionless model. We have used values given in Table 1 with $c_{\text{AI},0}^{\text{drop}}=10$ g/L. The one-factor-at-a-time method has been utilized to determine parameter sensitivity (Saltelli et al., 2000). Percent Ca uptake was calculated by dividing the final uptake given by Equation (27) by the initial applied concentration (5, 25, 50, 75, and 150 μg) × 100%. A selection of dimensioned and dimensionless parameters having the most to the least effect on percentage uptake at 48 h are shown in Table 2. The percentages given in Table 2 are a means of ranking the parameter sensitivities. The parameters in the table have been compared by calculating the ratio of the change in percentage uptake to the relative percentage change of the parameter at 48 h, namely,

$$\%\text{ Relative Parameter Sensitivity}=\frac{\Delta \;\%\text{ Uptake}}{\Delta \;\%\text{ Parameter}}.$$

**Table 2:** **Model parameters relative sensitivities**.

**Sensitivity**	**Parameter**	**% Relative parameter sensitivity**
Extreme	$\bar{F}$	133%
	ρ_0_	111%
High	*H*	102%
	*k*	100%
	*V*_0_	99%
Moderate	θ_0_	93%
	*b*	80%
	$\bar{\gamma }$	60%
Minute	$c_{\text{AI},0}^{\text{drop}}$	3%

*Relative sensitivity of parameters to percentage uptake at 48 h–highest to lowest level of sensitivity*.

#### 3.1.1. Fractal scaling dimension

**Figure 5:**
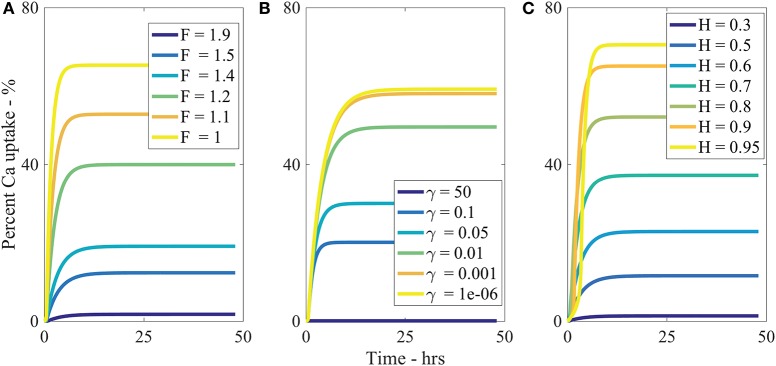
**Percent calcium (Ca) uptake sensitivity to dimensionless parameters $\bar{F}$ (A)**, $\bar{\gamma }$ **(B)**, and *H* **(C)** over 48 h with parameters described in Table 1.

The parameter $\bar{F}$ describes the tortuosity of the aqueous pores. It is based on the fractal scaling dimension, *F*_*s*_ and impacts the effective diffusivity of water and AI through the cuticle. We investigate how $\bar{F}$ influences uptake in Figure 5A and we see that increasing $\bar{F}$ decreases percentage uptake, as larger $\bar{F}$ produces more tortuous pores. As seen in Table 2, $\bar{F}$ has the most extreme effect over percentage uptake of all the parameters studied. Small changes in $\bar{F}$ have a large effect on the percentage uptake. At lower $\bar{F}$ values, the diffusivity function is increased and uptake can occur more rapidly. We expect that the tortuous nature of aqueous pores will differ significantly between plant species cuticles. Therefore, the extreme effect that $\bar{F}$ exhibits in the model would indicate that plant species variation itself has a significant effect on uptake. This is consistent with what is observed experimentally (Schreiber et al., 2006).

#### 3.1.2. Aqueous pore density

Increasing ρ_0_ increases percentage uptake and has the second most extreme effect on theoretical uptake as indicated in Table 2. As ρ_0_ increases, the number of pores under the drop increases, which in turn increases uptake. The pore density dictates the porosity of the cuticle.

We make the point that changing cuticle structures between plant species in nature would also be characterized by a change in the effective pore density. Like the discussion on $\bar{F}$, the high sensitivity of the model to this parameter could then explain why differences in plant species (Schreiber et al., 2006), plays a vital role in uptake.

#### 3.1.3. Relative humidity

The sensitivity of our model to relative humidity, *H*, can be seen in Figure 5C, which shows that increasing relative humidity increases percentage uptake. Relative humidity is highly influential over theoretical percentage uptake, as shown in Table 2. In the model, relative humidity influences uptake by affecting the evaporation and initial size of the aqueous pores.

In real cuticles, there is limited experimental data published investigating ionic uptake through cuticles without surfactant, at various humidities. Experiments are usually conducted with surfactants. However, other studies are worth considering. Several studies have shown relative humidity is directly proportional to percentage uptake of ionic AIs with surfactants through cuticles (Schönherr, 2000; Schönherr, 2001; Schönherr, 2002). These studies have also shown that the highest uptake always occurs at high relative humidity, where 90–100% produce similar results, which is also true in Figure 5C.

Middleton and Sanderson (1965) have investigated strontium-89, which is in the same periodic family as calcium and is known to metabolize in a similar fashion. Middleton and Sanderson (1965) find uptake in plant leaves is around 70% at relative humidity higher than 95% of strontium-89 after 50 h. Therefore, the maximum as shown in Figure 5C of 70% uptake at 95% relative humidity aligns with the well-established literature.

Our model predicts a 4-fold increase in percentage uptake when relative humidity is increased from 60 to 95%. Santier and Chamel (1992) apply glyphosate without surfactant to isolated tomato fruit CMs. They found a 9-fold increase in uptake when relative humidity increased from 60 to 100%. These results indicate our model has the potential to predict changes in relative humidity, beyond the Kraemer et al. (2009) data. Therefore, our results from the sensitivity analysis for relative humidity are feasible.

#### 3.1.4. Ion binding rate

Percentage uptake is affected by the ion binding rate, *k*. As *k* increases, percentage uptake decreases as more ions are bound to the cuticle surface and not available for diffusion. The parameter *k* highly affects percentage uptake, as shown in Table 2. If *k* is zero, the maximum uptake is reached (not shown). Moreover, the parameter *k* dramatically changes the shape of the uptake curve, that is the timescale to reach maximum uptake. The time to reach the maximum percentage uptake here ranges from 1 to 20 h. Yamada et al. (1964) also found ion binding effects dramatically altered the timescale to reach the maximum uptake for Ca^2+^ and Cl^−^ ions. The parameter *k* has the largest impact out of all the parameters studied on the time it takes to reach the maximum percentage uptake.

#### 3.1.5. Initial concentration of active ingredient

As shown in Table 2, the initial concentration of AI in the applied drop, $c_{\text{AI},0}^{\text{drop}}$, has very little effect on the final percentage uptake at 48 h. The parameter $c_{\text{AI},0}^{\text{drop}}$ affects the timescale to reach the maximum uptake. As the $c_{\text{AI},0}^{\text{drop}}$ increases, the time to reach the maximum uptake is longer, as $c_{\text{AI},0}^{\text{drop}}$ influences *V*_∞_, which effects the evaporation time of the drop.

#### 3.1.6. Other parameters

Several other parameters were studied as shown in Table 2. By decreasing θ_0_, *b*, or $\bar{\gamma }$ or increasing *V*_0_, percentage uptake is increased. The parameter θ_0_ influences the contact area of the droplet and the number of pores under the drop. Therefore, a low flat drop will cover more cuticle surface area and spread the AI over more aqueous pores, also causing the drop to stay on the leaf and not roll off, which is advantageous here. The parameter *b*, which is the thickness of the cuticle and can vary from 0.1 to 10 μm (Holloway, 1982; Jeffree, 1996), has an inverse relationship with percentage uptake. Ionic AIs take longer to diffuse through the pores of thicker cuticles, which is supported by the literature (Santier and Chamel, 1992). The parameter $\bar{\gamma }$ describes the volume of pores through the cuticle and is shown in Equation (29). It influences the surface droplet boundary condition in Equation (A-15) and how much AI can diffuse into the cuticle from the surface solution. In Figure 5B we can see an inverse relationship exists between $\bar{\gamma }$ and percentage uptake, which is due to a decreasing flux term in Equation (A-15).

Overall the results from the sensitivity analysis align with the well-established mechanisms influencing uptake of ionic AI through plant cuticles. By using this model over others previously presented in the literature, a mechanistic approach is achieved. We have simulated the important governing mechanisms in our model that affect ionic uptake in aqueous pores including relative humidity, plant species variation through tortuosity and density of aqueous pores, ion binding effects, concentration of AI, evaporation, droplet characteristics and variations in type of ionic AI used. Considering the results of the sensitivity analysis, $\bar{F}$ and ρ_0_ are the most influential over percentage uptake, indicating cuticle structure plays the most vital role in uptake. Also, *H*, *V*_0_ and *k* influence uptake substantially indicating climatic conditions and cuticle surface ion binding effects also play an important role in uptake.

## 4. Conclusion

A nonlinear, porous diffusion model has been developed here to simulate diffusion of hydrophilic ionic AI and diffusion with adsorption of water through a plant cuticle. This model makes novel additions to a simple diffusion model by incorporating the swelling of aqueous pores with water, climatic conditions such as relative humidity that affect the evaporation of water in the applied droplet, parameters that account for differences between plant species, porosity and tortuosity of the aqueous pores, cuticle surface ion binding and a diffusivity function that changes through the cuticle over time. The nonlinear model has been solved numerically, producing results that show good agreement with experimental data. Major factors influencing our model's uptake of ionic AI through plant cuticles have been found to be cuticle structure, including tortuosity and density of the aqueous pores, and to a lesser extent (while still high), humidity and cuticle surface ion binding effects through the sensitivity analysis.

## Author contributions

ET is responsible for article writing, model creation and adaptation, computational code creation, results, analysis, article editing and revision. TF is responsible for model creation and adaptation, results, analysis, article editing and revision. WF is responsible for motivating the project, the biological and agrochemical consultation, article editing and revision. SP is responsible for computational code editing, article editing and revision. All authors agree to be accountable for all aspects of the work in ensuring that questions related to the accuracy or integrity of any part of the work are appropriately investigated and resolved.

### Conflict of interest statement

The authors declare that the research was conducted in the absence of any commercial or financial relationships that could be construed as a potential conflict of interest.

## Footnotes

^1^http://www.glchloride.com/brochure/Brochure-Calciumchloridehandbook.pdf
