# Supplementary material for: Nonlinear Porous Diffusion Modeling of Hydrophilic Ionic Agrochemicals in Astomatous Plant Cuticle Aqueous Pores: A Mechanistic Approach
Source: Front Plant Sci. 2017 May 10;8:746. doi: 10.3389/fpls.2017.00746 (PMC5423917; doi:10.3389/fpls.2017.00746)
Supplement: Supplementary file 1 [file DataSheet1.pdf]

A Dimensionless Model

The full dimensionless model is described here, where Equations (A-1) describe the dimensionless parameters and Equations (A-2) to (A-19) describes the dimensionless model created using these parameters.

By making the following substitutions:

$$\left.\begin{aligned}
&\bar{x} = \frac{x}{b}, \quad \bar{t} = \frac{D_{\mathrm{AI}}^{\mathrm{bulk}} t}{b^2}, \quad \overline{c_1} = \frac{c_{\mathrm{AI}}}{c_{\mathrm{AI,0}}^{\mathrm{drop}}}, \quad \overline{c_2} = \frac{c_{\mathrm{H_2O}}}{c_{\mathrm{H_2O}}^{\mathrm{pure}}}, \quad \overline{D}_1 = \frac{D_{\mathrm{AI}}}{D_{\mathrm{AI}}^{\mathrm{bulk}}},\\
&\overline{D}_2 = \frac{D_{\mathrm{H_2O}}}{D_{\mathrm{H_2O}}^{\mathrm{bulk}}}, \quad \overline{D}_{\mathrm{total}} = \frac{D_{\mathrm{H_2O}}^{\mathrm{bulk}}}{D_{\mathrm{AI}}^{\mathrm{bulk}}}, \quad \bar{r} = \frac{r_{\mathrm{p}}}{r_{\mathrm{H_2O}}}, \quad \overline{\Gamma} = \frac{\Gamma_{\mathrm{H_2O}}}{\Gamma_{\mathrm{S}}},\\
&\overline{\beta} = \beta_{\mathrm{H_2O}}\, c_{\mathrm{H_2O}}^{\mathrm{pure}}, \quad \Omega = \Gamma_{\mathrm{S}}\, r_{\mathrm{H_2O}}^2\, N_{\mathrm{A}}, \quad \overline{V} = \frac{V}{V_0}, \quad \overline{F} = \frac{F_s}{2 - F_s},\\
&\overline{v}_1 = \overline{v}_{\mathrm{AI}}\, c_{\mathrm{AI,0}}^{\mathrm{drop}}, \quad \overline{v}_2 = \overline{v}_{\mathrm{H_2O}}\, c_{\mathrm{H_2O}}^{\mathrm{pure}}, \quad \Psi = \frac{\delta r_{\mathrm{drop}}^3}{V_0}, \quad \overline{E} = \frac{2\, D_{\mathrm{evap}}\, \psi\, b^2}{r_{\mathrm{drop}}^2\, \rho_{\mathrm{L}}\, D_{\mathrm{AI}}^{\mathrm{bulk}}},\\
&\omega = \frac{k\, b^2}{D_{\mathrm{AI}}^{\mathrm{bulk}}\, V_0}, \quad \mu = \frac{2\, \Gamma_{\mathrm{S}}}{r_{\mathrm{H_2O}}\, c_{\mathrm{H_2O}}^{\mathrm{pure}}}, \quad \overline{\gamma} = \frac{\rho_0\, A_{\mathrm{drop}}\, A_{\Pi}\, b}{V_0}, \quad \Lambda = \frac{V_0}{\delta r_{\mathrm{drop}}^3 + \frac{V_0}{4}},
\end{aligned}\right\} \tag{A-1}$$

Equations (1) to (23) can be expressed in dimensionless form as:

PDEs :

$$\frac{\partial(\varepsilon \overline{c_1})}{\partial \bar{t}} = \frac{\partial}{\partial \bar{x}}\left[\overline{D}_1 \left(\frac{\partial(\varepsilon \overline{c_1})}{\partial \bar{x}}\right)\right], \qquad 0 < \bar{x} < 1,\ \bar{t} > 0, \tag{A-2}$$

$$\frac{\partial(\varepsilon \overline{c_2})}{\partial \bar{t}} = \overline{D}_{\mathrm{total}} \frac{\partial}{\partial \bar{x}}\left[\overline{D}_2 \left(\frac{\partial(\varepsilon \overline{c_2})}{\partial \bar{x}}\right)\right] - \frac{\mu}{\bar{r}}(1-\varepsilon)\frac{\partial \overline{\Gamma}}{\partial \bar{t}}, \quad 0 < \bar{x} < 1,\ \bar{t} > 0, \tag{A-3}$$

Functions : $$\bar{\Gamma}(\bar{x},\bar{t}) = \left(1 + \left(\bar{\beta}\bar{c_2}\right)^{-1}\right)^{-1}, \qquad 0 < \bar{x} < 1,\ \bar{t} > 0, \qquad \text{(A-4)}$$

$$\bar{r}(\bar{x},\bar{t}) = 1 + \left(\sin((\Omega\bar{\Gamma})^{-1})\right)^{-1}, \qquad 0 < \bar{x} < 1,\ \bar{t} > 0, \qquad \text{(A-5)}$$

$$\varepsilon(\bar{x},\bar{t}) = \pi\left[\frac{\bar{r}\ r_{\mathrm{H_2O}}}{L}\left(\sqrt{n_0} + 1\right)\right]^2, \qquad 0 < \bar{x} < 1,\ \bar{t} > 0, \qquad \text{(A-6)}$$

$$\overline{D}_1(\bar{x},\bar{t}) = \overline{D}_2(\bar{x},\bar{t}) = \varepsilon^{\overline{F}}, \qquad 0 < \bar{x} < 1,\ \bar{t} > 0, \qquad \text{(A-7)}$$

ICs : $$\bar{c_1}(\bar{x},0) = 0, \qquad 0 < \bar{x} < 1, \qquad \text{(A-8)}$$

$$\bar{c_1}(0,0) = 1, \qquad \text{(A-9)}$$

$$\bar{r}(\bar{x},0) = \frac{r_{\mathrm{p}}^{\mathrm{max}}}{r_{\mathrm{H_2O}}}\ H, \qquad 0 \le \bar{x} \le 1, \qquad \text{(A-10)}$$

$$\bar{c_2}(\bar{x},0) = 1, \qquad 0 < \bar{x} < 1, \qquad \text{(A-11)}$$

$$\bar{c_2}(0,0) = \frac{1 - \bar{v}_1\bar{c_1}(0,0)}{\bar{v}_2}, \qquad \text{(A-12)}$$

$$\bar{\Gamma}(\bar{x},0) = \left(\Omega \arcsin\left((\bar{r}(\bar{x},0) - 1)^{-1}\right)\right)^{-1}, \qquad 0 < \bar{x} < 1, \qquad \text{(A-13)}$$

$$\bar{\beta} = \left(\bar{c_2}(\bar{x},0)\left[\frac{1}{\bar{\Gamma}(\bar{x},0)} - 1\right]\right)^{-1}, \qquad 0 < \bar{x} < 1, \qquad \text{(A-14)}$$

BCs - AI (drop) : $$\frac{\partial}{\partial\bar{t}}\left[\overline{V}\ \bar{c_1}(0,\bar{t})\right] = -\omega\ \bar{c_1}(0,\bar{t}) + \bar{\gamma}\ \overline{D}_1(0,\bar{t})\frac{\partial}{\partial\bar{x}}\left[\varepsilon\ \bar{c_1}\right]\Big|_{\bar{x}=0}, \qquad \text{(A-15)}$$

$$\overline{V}(\bar{t}) = \begin{cases} \dfrac{\Psi}{\left(\Lambda - \overline{E}\bar{t}\right)^{-1} - \frac{1}{4}} & : \overline{V}(\bar{t}) > \dfrac{V_\infty}{V_0}, \\ \dfrac{V_\infty}{V_0} & : \overline{V}(\bar{t}) \le \dfrac{V_\infty}{V_0}, \end{cases} \qquad \text{(A-16)}$$

BC - AI (bath) : $$\bar{c_1}(1,\bar{t}) = 0, \qquad \bar{t} > 0, \qquad \text{(A-17)}$$

BC - H_2O (drop) : $$\bar{c_2}(0,\bar{t}) = \frac{1 - \bar{v}_1\bar{c_1}(0,\bar{t})}{\bar{v}_2}, \qquad \bar{t} > 0, \qquad \text{(A-18)}$$

BC - H_2O (bath) : $$\bar{c_2}(1,\bar{t}) = 1, \qquad \bar{t} > 0. \qquad \text{(A-19)}$$
